# Drug-induced kidney injury: challenges and opportunities

**DOI:** 10.1093/toxres/tfae119

**Published:** 2024-08-05

**Authors:** Skylar Connor, Ruth A Roberts, Weida Tong

**Affiliations:** National Center for Toxicological Research, US Food and Drug Administration, Jefferson, AR 72079, United States; ApconiX Ltd, Alderley Park, Alderley Edge, SK10 4TG, United Kingdom; University of Birmingham, Edgbaston, Birmingham B15 2TT, United Kingdom; National Center for Toxicological Research, US Food and Drug Administration, Jefferson, AR 72079, United States

**Keywords:** renal injury, drug discovery, nephrotoxicity, DIKI, kidney injury

## Abstract

Drug-induced kidney injury (DIKI) is a frequently reported adverse event, associated with acute kidney injury, chronic kidney disease, and end-stage renal failure. Prospective cohort studies on acute injuries suggest a frequency of around 14%–26% in adult populations and a significant concern in pediatrics with a frequency of 16% being attributed to a drug. In drug discovery and development, renal injury accounts for 8 and 9% of preclinical and clinical failures, respectively, impacting multiple therapeutic areas. Currently, the standard biomarkers for identifying DIKI are serum creatinine and blood urea nitrogen. However, both markers lack the sensitivity and specificity to detect nephrotoxicity prior to a significant loss of renal function. Consequently, there is a pressing need for the development of alternative methods to reliably predict drug-induced kidney injury (DIKI) in early drug discovery. In this article, we discuss various aspects of DIKI and how it is assessed in preclinical models and in the clinical setting, including the challenges posed by translating animal data to humans. We then examine the urinary biomarkers accepted by both the US Food and Drug Administration (FDA) and the European Medicines Agency for monitoring DIKI in preclinical studies and on a case-by-case basis in clinical trials. We also review new approach methodologies (NAMs) and how they may assist in developing novel biomarkers for DIKI that can be used earlier in drug discovery and development.

## Introduction

Kidney disease is the 10^th^ leading cause of death in the United States (U.S.), contributing to 54,358 deaths in 2021 alone, with nephritis, nephrotic syndrome, and nephrosis accounting for this mortality.^1^ While nephrotoxicity comprises a wide spectrum of diseases, here we define it as the rapid deterioration of kidney function or kidney injury due to the damaging and toxic effects of drugs, chemicals, and toxins.^2^ Around 20% of nephrotoxicity cases are attributed to drugs.^2^ Drug-induced kidney injury (DIKI) can lead to the development of acute kidney injury (AKI), chronic kidney disease (CKD), or end-stage renal disease, causing over 1.5 million adverse events annually and affecting approximately 26% of the U.S. population.^3^^,^^4^ Prospective cohort studies on acute injury suggest a frequency of around 14%–26% in adult populations and a significant concern in pediatrics, where 16% of acute injury cases are attributed to drug-induced causes (see Awdishu and Mehta 2017^5^ for a detailed review of incidence).

**Fig. 1:**
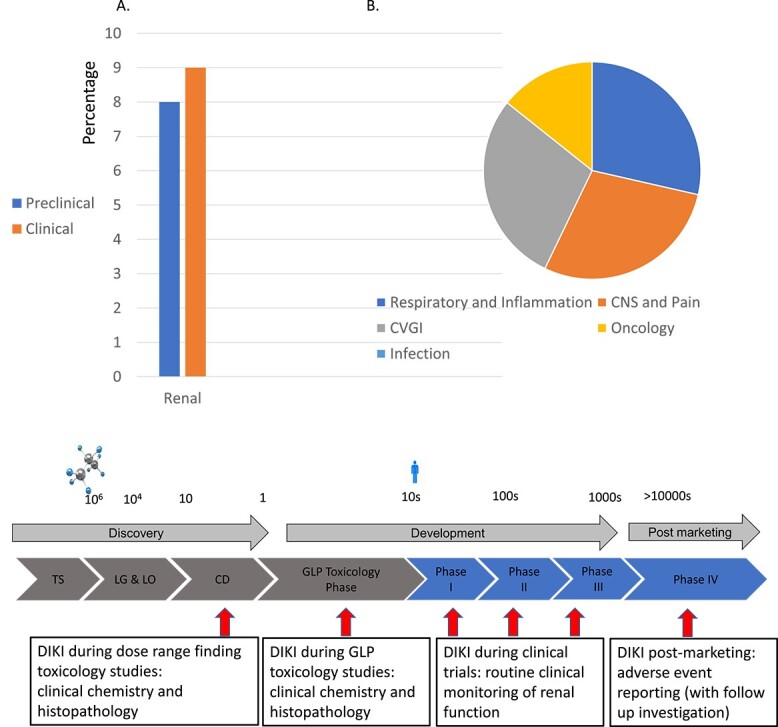
A) Incidence of preclinical and clinical failures due to renal injury. B) Incidence of project closure due to renal injury by therapy area. See Cook et al. 2014^6^ for original data.

In addition to the impact on patients and the healthcare system, drug failures due to DIKI is a major concern for the pharmaceutical industry, given its frequently reported occurrence in drug discovery and development. Specifically, safety/toxicity-related failures account for 82% of drug project closures,^6^ and among these, renal injury accounts for 8% and 9% of preclinical and clinical failures, respectively (Fig. 1A). DIKI spans various therapeutic areas, including respiratory/inflammation, cardiovascular/gastrointestinal, and central nervous system (CNS)/pain (Fig. 1B). Notably, of interest to DIKI, only 3.6% of urology drugs and even fewer renal specific drugs progress from phase I to approval in clinical trials.^7^ This suggests that the kidney has a higher susceptibility to drug-induced injury, as it is exposed to higher concentrations of circulating drugs and/or metabolites, as compared to other organ systems.^8^^,^^9^ There are several factors that contribute to the accumulation of nephrotoxins within the kidney, such as its high vascularity (receiving about 25% of resting cardiac output) and the gradual increase in the concentration of intraluminal nephrotoxins through the reabsorption of the glomerular filtrate.^9^

## Current approaches for assessing DIKI

DIKI is assessed preclinically in good laboratory practice (GLP) studies as specified in the International Council for Harmonisation of Technical Requirements for Pharmaceuticals for Human Use (ICH) guidelines.^10^ Typically, these studies span 28 days in one rodent and one non-rodent species and may include a recovery period. Within these in vivo studies, DIKI may be detected by altered renal histopathology and/or changes in clinical chemistry endpoints, such as serum creatinine (sCr) or blood urea nitrogen (BUN).^11^ Similarly, in humans, renal injury is diagnosed using sCr and BUN, historically standard biomarkers for renal function. One advantage of these biomarkers is their functionality, as healthy kidneys should filter them out of the blood into urine. However, they have limitations in detecting human renal injury, as they are influenced by many renal and non-renal factors independent of kidney function such as age, sex, muscle mass, use of supplements, ingestion of cooked meats, medication, and frequency of intense resistance training and exercise.^8^ Given the inability of diagnostic methods, like sCr and BUN, to detect the initial stages of renal injury and distinguish between DIKI and other types of AKI, many investigators and regulatory agencies are advocating for new approach methods (NAMs) and more sensitive biomarkers. These would aid in eliminating new drug candidates with unfavorable risk–benefit profiles at an earlier stage and with greater accuracy.^12^ Within the scope of DIKI, several blood/serum and urinary biomarkers have been widely accepted in clinical practice and by governing agencies, (Table 1).

**Table 1:** Historic and FDA approved biomarkers for nephrotoxic safety.

**Biomarker**	**Source/ Medium**	**Notes**	**Reference**
Albumin (albuminuria)	Urine	FDA- approved urinary nephrotoxicity safety biomarker intended to be used in the context of nonclinical drug development for the detection of acute drug-induced nephrotoxicity. Intended to identify acute drug-induced changes in renal tubules in rats Can complement conventional clinical chemistry markers and histopathology in GLP toxicology studies conducted during clinical trials to assess renal safety. Most abundant plasma protein Commonly employed in clinical settings as an indicator of kidney impairment among diabetic patients and as a measure of end-stage renal disease in individuals with hypertension. Can be found in the urine of patients with glomerular or tubular kidney injury, but much higher levels of urinary albumin are usually observed with glomerular injury than with tubular injury, tubular injury generally shows less than 500 mg/24 h of albumin excretion. Nonclinical urinary nephrotoxicity safety biomarkers accepted by the EMEA and Japanese PMDA for use in rat GLP studies	^8^ ^,^ ^13–17^
Beta-2 Microglobulin (B2M)	Urine	FDA- approved urinary nephrotoxicity safety biomarker intended to be used in the context of nonclinical drug development for the detection of acute drug-induced nephrotoxicity. Intended to detect acute drug-induced glomerular changes or damage and/or impairment of tubular reabsorption in the kidney of rats Can complement conventional clinical chemistry markers and histopathology in GLP toxicology studies conducted during clinical trials to assess renal safety. One of the most commonly utilized urinary proteins for monitoring cisplatin-induced AKI in patients. Levels of urinary B2M are known to rise following the administration of several nephrotoxic agents, such as cisplatin, cyclosporine, and gentamicin. Based on current data the correlation between short-term B2M rise and long-term GFR reduction continues to remain unclear. Nonclinical urinary nephrotoxicity safety biomarkers accepted by the EMEA and Japanese PMDA for use in rat GLP studies	^8^ ^,^ ^13–18^
Blood Urea Nitrogen (BUN)	Blood	Historically used to diagnose AKI One of the standard safety biomarker currently used to monitor kidney function and drug-induced renal toxicity. A marker for kidney function Used to help diagnose or monitor a kidney disease or disorder. A waste product removed from the blood by the kidneys Known as a poor predictor of drug-induced renal damage due to the lack sensitivity and specificity for renal injury Does not detect early or minimal kidney injury Increase in marker is not seen until after a significant compromise of kidney function has occurred	^8^ ^,^ ^13^ ^,^ ^14^ ^,^ ^19^ ^,^ ^20^
Clusterin (CLU)	Urine	FDA- approved urinary nephrotoxicity safety biomarker intended to be used in the context of nonclinical drug development for the detection of acute drug-induced nephrotoxicity. Intended to detect acute drug-induced kidney tubular alterations in rats Can complement conventional clinical chemistry markers and histopathology in GLP toxicology studies conducted during clinical trials to assess renal safety. One of six FDA-approved urinary safety biomarkers included in the safety composite biomarker panel. Its purpose is to complement traditional measures and assist in identifying kidney tubular injury during phase 1 trials conducted with healthy volunteers. Expressed in response to injury Notably abundant during the initial phases of kidney development and is subsequently upregulated after glomerular, tubular, and papillary injuries in animals. Performs well in the detection of proximal tubule injury, including cisplatin or gentamicin induced nephrotoxicity. Suggested to be one of the earliest markers of proximal tubular injury. Nearly all data on clusterin is derived from animal models, human clinical short and long-term data has not been adequately studied Nonclinical urinary nephrotoxicity safety biomarkers accepted by the EMEA and Japanese PMDA for use in rat GLP studies	^8^ ^,^ ^13–18^ ^,^ ^21–23^
Cystatin C (CysC)	Urine, Serum	FDA- approved urinary nephrotoxicity safety biomarker intended to be used in the context of nonclinical drug development for the detection of acute drug-induced nephrotoxicity. Intended to detect acute drug-induced glomerular changes or damage and/or impairment of tubular reabsorption in the kidney of rats Can complement conventional clinical chemistry markers and histopathology in GLP toxicology studies conducted during clinical trials to assess renal safety. One of six FDA-approved urinary safety biomarkers included in the safety composite biomarker panel. Its purpose is to complement traditional measures and assist in identifying kidney tubular injury during phase 1 trials conducted with healthy volunteers. Freely filtered, reabsorbed, and metabolized by tubules Urinary cystatin C is an early biomarker of ischemic AKI and nephrotoxicity in humans Serum cystatin C has demonstrated a better predictive capability for vancomycin-induced nephrotoxicity in comparison to serum creatinine. Consequently, it has been integrated into vancomycin drug-dosing algorithms to enhance medication safety. Both serum and urinary cystatin C show mixed results in the detection of cisplatin-induced nephrotoxicity, but has the ability to effectively predict AKI induced by aminoglycosides, amphotericin B, radiocontrast dye, high dose methotrexate, and vancomycin. Nonclinical urinary nephrotoxicity safety biomarkers accepted by the EMEA and Japanese PMDA for use in rat GLP studies	^8^ ^,^ ^13–18^ ^,^ ^21^ ^,^ ^22^ ^,^ ^24^
Serum Creatinine (sCr)	Serum	A small molecule generated in muscle that can serve as a functional marker, historically used to diagnose AKI. Most widely used functional biomarker for the kidney Used to check how well your kidneys are filtering your blood Accumulation due to diminished renal excretion; predominantly influenced by glomerular rather than tubular injury One of the standard safety biomarker currently used to monitor kidney function and toxicity. Known as a poor predictor of drug-induced renal damage due to the lack sensitivity and specificity for renal injury Increase in marker is not seen until after a significant compromise of kidney function has occurred	^8^ ^,^ ^13^ ^,^ ^18^ ^,^ ^19^ ^,^ ^24–26^
Kidney Injury Molecule-1 (KIM-1)	Urine	FDA- approved urinary nephrotoxicity safety biomarker intended to be used in the context of nonclinical drug development for the detection of acute drug-induced nephrotoxicity. Intended to detect acute drug-induced kidney tubular alterations in rats Can complement conventional clinical chemistry markers and histopathology in GLP toxicology studies conducted during clinical trials to assess renal safety. One of six FDA-approved urinary safety biomarkers included in the safety composite biomarker panel. Its purpose is to complement traditional measures and assist in identifying kidney tubular injury during phase 1 trials conducted with healthy volunteers. Expressed in response to injury Proteolytically processed domain detected in urine after injury Suggested to be one of the earliest markers of proximal tubular injury. An early biomarker of ischemic AKI in humans. highly upregulated in late-stage AKI Nonclinical urinary nephrotoxicity safety biomarkers accepted by the EMEA and Japanese PMDA for use in rat GLP studies	^8^ ^,^ ^13–18^ ^,^ ^21^ ^,^ ^22^ ^,^ ^24^ ^,^ ^27^
N-acetyl-beta-D-glucosaminidase (NAG)	Urine	One of six FDA-approved urinary safety biomarkers included in the safety composite biomarker panel. Its purpose is to complement traditional measures and assist in identifying kidney tubular injury during phase 1 trials conducted with healthy volunteers. Cisplatin administration in rats caused an increase in urinary NAG activity Experiments have indicated that NAG is a sensitive marker of acute oxidative stress within the kidney regardless of development of acute kidney injury	^13^ ^,^ ^18^ ^,^ ^22^
Neutrophil Gelatinase-Associated Lipocalin (NGAL)	Urine, Serum	One of six FDA-approved urinary safety biomarkers included in the safety composite biomarker panel. Its purpose is to complement traditional measures and assist in identifying kidney tubular injury during phase 1 trials conducted with healthy volunteers. Expressed at low levels in various tissues with upregulated transcription in tubuloepithelial cells following ischemic and nephrotoxic kidney injuries. Significantly increased in injured tubular (especially proximal renal tubular) epithelial cells after renal ischemia or toxic damage A prospective study has found urinary NGAL to be a powerful early marker of AKI	^8^ ^,^ ^13^ ^,^ ^18^ ^,^ ^21^ ^,^ ^22^ ^,^ ^24^
Osteopontin (OPN)	Urine	One of six FDA-approved urinary safety biomarkers included in the safety composite biomarker panel. Its purpose is to complement traditional measures and assist in identifying kidney tubular injury during phase 1 trials conducted with healthy volunteers. Increased mRNA and protein levels of OPN have been seen after kidney injury OPN expression is increased in the kidneys, blood, and urine of patients with CKD, particularly in those with diabetic kidney disease and glomerulonephritis.	^13^ ^,^ ^22^ ^,^ ^28^ ^,^ ^29^
Renal Papillary Antigen (RPA-1)	In vivo tissue, Urine	FDA- Approved Safety biomarker to be used with traditional indicators to indicate renal injury in rat Intended for voluntary use as a urinary biomarkers of drug-induced kidney toxicity in male rat safety assessment studies for the detection of acute drug-induced collecting duct injury, when used in conjunction with traditional clinical chemistry markers and histopathology in GLP toxicology studies Found to have high specificity in detecting injury to collecting duct cells over other tubular cell types in rats. Can be detected in urine at early stages of toxicity Largely kidney specific Found in urine early after damage, at a stage where renal papillary necrosis is not histopathologically present and is potentially reversible.	^13^ ^,^ ^23^ ^,^ ^30^ ^,^ ^31^
Total Protein (Proteinuria, albuminuria)	Urine	FDA- approved urinary nephrotoxicity safety biomarker intended to be used in the context of nonclinical drug development for the detection of acute drug-induced nephrotoxicity. Intended to detect acute drug-induced glomerular changes or damage and/or impairment of tubular reabsorption in the kidney of rats Can complement conventional clinical chemistry markers and histopathology in GLP toxicology studies conducted during clinical trials to assess renal safety. Commonly utilized in clinical practice as an indicator of kidney damage to monitor disease progression and assess the effectiveness of therapy Can be detected in the urine of patients experiencing glomerular or tubular kidney injury, although higher levels of urinary albumin are usually observed with glomerular injury The roles of albuminuria and proteinuria as drug-induced nephrotoxicity markers has been most thoroughly investigated in patients receiving cisplatin. The impact of cyclosporine on proteinuria levels has been investigated in both animals and humans. Urinary albumin is observed in higher levels with glomerular injury than with tubular injury Nonclinical urinary nephrotoxicity safety biomarkers accepted by the EMEA and Japanese PMDA for use in rat GLP studies	^8^ ^,^ ^13–17^
Trefoil Factor 3 (TFF3)	Urine, Serum	FDA- approved urinary nephrotoxicity safety biomarker intended to be used in the context of nonclinical drug development for the detection of acute drug-induced nephrotoxicity. Intended to detect acute drug-induced kidney tubular alterations in rats Can complement conventional clinical chemistry markers and histopathology in GLP toxicology studies conducted during clinical trials to assess renal safety. TFF3 levels are significantly increased in the serum and urine of individuals with chronic kidney disease (CKD) TFF3 displays potential as a prognostic marker in both clinical and subclinical chronic kidney disease (CKD). However, there is limited understanding regarding its performance in relation to nephrotoxicity Nonclinical urinary nephrotoxicity safety biomarkers accepted by the EMEA and Japanese PMDA for use in rat GLP studies	^8^ ^,^ ^13–17^ ^,^ ^21^

**Table 2:** A list of NAMs currently used in the field of DIKI.

New Approach Method (NAM)	Notes	Reference
TIMP2 AND IGFBP7 (NephroCheck®)	The first platform approved by the FDA Marketed as a biomarker of AKI Approved for use is currently limited to critically ill patients Detects kidney stress in patients at risk for AKI. Intended to be used in conjunction with clinical evaluations as an aid to support the risk assessment of moderate or severe AKI in acutely ill patients. Has not conclusively predicted nephrotoxicity in stable patients Assists in facilitating the early identification of AKI in critically ill patients, allowing time for reduced dosing of known nephrotoxic medications prior to injury	^8^ ^,^ ^21^ ^,^ ^39–42^
Kidney-on-a-Chip	in vitro microphysiological system (MPS) Reproduces a 3D microenvironment Microfluidic platforms designed to replicate the structural and functional properties of the kidney Integrated with living cells Mimics the structural, mechanical, transport, absorptive, and physiological properties of the human kidney Enables high-resolution and real-time molecular imaging of complex in vitro systems More physiologically relevant as compared to 2D standard cultures Subjected to mechanical stimuli such as flow	^13^ ^,^ ^37^ ^,^ ^43–46^
Functional Nephron Number	Considered an important determinant of kidney health and disease susceptibility throughout life Has the potential to be used as a clinical biomarker, where it can provide vital information regarding. . . the progression of kidney disease provide early detection of CKD onset provide assessment of recovery after AKI predict the risk of drug-induced nephrotoxicity, assist in the development of strategies for dosing and toxicity testing for a wide range of therapeutic drugs	^37^
RNA Biomarkers	There are several examples of the potential use of microRNAs (miRNA) in the context of drug-induced kidney injury (DIKI) A cross-laboratory program identified urinary miRNA patterns associated with cell- or cause-specific DIKI characterized biomarkers in rats from different nephron regions. Proving urinary miRNA panels from different nephron regions may contribute to the identification of the DIKI potential of novel drugs	^37^ ^,^ ^47^
Stem Cell Therapy	Alternative approach on the rise in the last few decades Protects from cisplatin-induced nephrotoxicity, AKI, diabetic nephropathy, and oxidative stress through the activation of autophagy	^37^
QSAR Modeling	Quantitative structure–activity relationship (QSAR) While QSAR modelling has been applied for the last 6 decades, pairing it with machine learning (ML) and deep learning (DL) modeling techniques is fairly new (appearing within the last decade) There is evidence that “deep QSAR” methods (termed by Tropsha et al.) have accelerated the preclinical research stages for small-molecule drug candidates. There has been a number of QSAR models built for renal toxicity using ML and DL methods like: regression modelling, naïve Bayes (NB), associative neural network (ASNN) support vector machine (SVM), random forest (RFR), extreme gradient boosting (XGBoost), C4.5 decision tree, convolutional neural network fingerprint (CNF), transformer convolutional neural network (TRANSNN), and Graph Isomorphism Network (GIN) to name a few	^4^ ^,^ ^48–50^

Blood/serum origin biomarkers such as sCr and BUN tend to have a long history of research, are highly validated, have been well established for certain disease states, and are less influenced by diet and hydration making them more stable as compared to urinary biomarkers.^32^ While blood/serum has a number of advantages such as convenience and use in general routine testing, they may only show significant elevations after substantial renal injury has already occurred.^8^^,^^32^ Research has found that urinary biomarkers have the ability to outperform serum markers for certain diseases.^32^ The advantages of using urinary biomarkers include non-invasive and repeated collection, allowing for long-term monitoring, aiding in early detection. They may also reflect proximal changes in organ function and damage. However, the disadvantages include their limited reflection of different disease areas, the sample variability due to diet or hydration, and the need for further research and validation for clinical application.^32^

## Translating animal results to human risk for DIKI

Establishing the relevancy of toxicity findings in animal studies to humans remains a challenge.^12^ Animal models, despite being extensively utilized, do not reliably translate to human toxicity nor do they accurately predict adverse events in clinical trials. This discrepancy may be attributed to the limited genetic diversity in laboratory animals.^12^^,^^33^ Aside from GLP studies that are typically conducted in rodent and non-rodent species, the vast majority of experimental work on DIKI is carried out in rodent models, even though the heterogenicity observed in mouse and rodent models does not fully mirror the complexity of what is seen in humans.^34^ Additionally, researchers may select a single species, gender, and age for experiments,^34^ which can introduce several limitations when translating animal data to human risk assessments. Typically, single causative agents are studied in animal models, while humans tend to experience numerous factors that lead to injury. Additionally, animal studies are often conducted in young adults, although it is elderly humans who are at the greatest risk for kidney injury.^35^ Overall, human renal injury is still poorly researched as compared to other organ systems such as the liver.

Currently, there are a number of promising nephrotoxicity biomarkers, such as urinary markers kidney injury molecule-1 (KIM-1), β2-microglobulin (B2M), cystatin C, clusterin, and trefoil factor-3 (TFF-3) (Table 1). These have been accepted as highly sensitive and specific urinary biomarkers by both the US Food and Drug Administration (FDA) and the European Medicines Agency for monitoring DIKI in preclinical studies and on a case-by-case basis in clinical trials.^2^^,^^8^^,^^36^ These accepted biomarkers span a broad range of roles in detecting disease progression. For example, KIM-1, NGA, and CLU biomarker are used in early detection and sCr, BUN, Kim-1, and albuminuria^37^ are used in late detection. Despite demonstrating considerable potential, the correlations between the rise and fall of these biomarkers and the subsequent development of clinically significant nephrotoxicity warrants further research.^8^

## New approach methods (NAMs) in the detection of DIRI

NAMs are methods developed to reduce, refine, or replace animal testing.^38^ NAMs offer the potential to be faster, less expensive, and more informative than current approaches for toxicological assessment. The FDA’s Center for Drug Evaluation and Research (CDER) defines NAMs broadly, encompassing in vitro, in chemico, and in silico methods.^12^ While it is unlikely that researchers and regulatory agencies will completely replace whole animal general toxicity studies in drug development,^12^ the development, validation, and adoption of NAMs provide an opportunity to reduce the number of animals used in testing, refine current methods that still require animals, and replace animal testing whenever possible.^38^ Currently, there are several new techniques and models, such as microphysiological systems (MPS), quantitative structure activity relationship (QSAR) computer-based models, and in vitro/in silico toxicity prediction tools, being studied and reported in the literature (Table 2). Most recently, there has been a breakthrough in the area of in silico or AI-driven drug discoveries, with companies like Exscientia reporting the first AI-designed drug candidate to enter clinical trials in 2020 and Insilico Medicine reporting a novel AI-designed first-in-class anti-fibrotic drug candidate for a novel target entering Phase I clinical trial in 2021.^48^ These innovations and novel methods may have utility in improving pre-clinical drug development programs for human risk,^12^ including the assessment of DIKI.

## The role of the DIRIL database in NAM research

The development and facilitation of NAMs for the study of DIKI could be expedited and enhanced by a highly annotated list of drugs with DIKI potential. The creation of DIRIL (drug induced renal injury list)^51^ will provide the opportunity for such an approach since DIRIL is a highly curated collection of single-molecule, oral admission drugs for human use. The aim of DIRIL is to serve as a research tool for the development and refinement of NAMs specific to DIRI. Using a binary (positive versus negative) classification system linked to compound therapeutic application, DIRIL provides an invaluable resource for research and development in nephrotoxicity. It is particularly relevant for enhancing the discovery of new methodologies to assess severity and better classify nephrotoxicity.

## Future perspectives

**Fig. 2:**
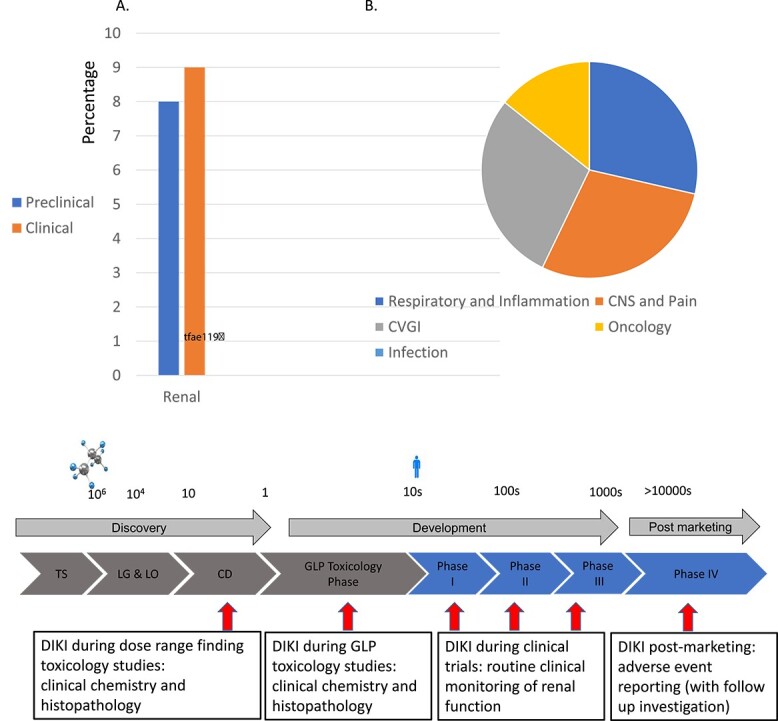
The potential application of biomarkers of DIKI during drug discovery and development. TS: Target selection; LG&LO: Lead generation and lead optimization; CD: Candidate drugs; DIKI: Drug-induced kidney injury; GLP: Good laboratory practice.

There is a pressing need for new markers to identify and gauge the severity of DIKI at various stages in drug discovery, development, and marketing. Such biomarkers could prove beneficial in dose range toxicology studies, facilitating the transition from discovery to development, or later during GLP toxicology studies (Fig. 2). Alternatively, they could be used to assess DIKI during clinical trials or even much later during the post marketing phases (Phase IV); biomarkers particularly from metabolomics research could be very powerful in the clinical setting and urinary biomarkers of metabolites have been found to provide a valuable perspective into the various physiological and pathological processes.^32^ Research has also indicated that renal biomarkers hold promise in genomic and mechanistic studies for a better understanding of AKI, which could aid in future drug development.^32^^,^^52^ The potential impact of such biomarkers for DIKI differs by phase; early preclinical detection of DIKI would allow for the redesign or avoidance of nephrotoxic compounds or drugs. The detection of DIKI later during clinical trials could aid in dose escalation and patient selection; and finally, detection during Phase IV would be invaluable in monitoring adverse events in a wider population. Over the last decade the field of DIKI research has been rapidly evolving, while there are a number of challenges and uncertainties there is also an abundance of opportunities.
